# G-Quadruplex Guanosine Gels and Single Walled Carbon Nanotubes

**DOI:** 10.3390/molecules181215434

**Published:** 2013-12-11

**Authors:** Yuehua Yu, Victor L. Pushparaj, Omkaram Nalamasu, Linda B. McGown

**Affiliations:** 1 Department of Chemistry and Chemical Biology, Rensselaer Polytechnic Institute, Troy, NY 12180, USA; 2 Department of Materials Science and Engineering, Rensselaer Polytechnic Institute, Troy, NY 12180, USA

**Keywords:** G-quadruplex, guanosine gel, guanosine tetrad, guanosine monophosphate, single-walled carbon nanotubes

## Abstract

Solubilization of single walled carbon nanotubes (SWNTs) in aqueous gel phases formed by reversible, G-quadruplex self-assembly of guanosine monophosphate (GMP) alone or with guanosine (Guo) is described. Unlike other media and methods for aqueous solubilization of SWNTs, the guanosine gels (“G-gels”) are found to readily disperse high (>mg/mL) concentrations of individual rather than bundled SWNTs. SWNT dispersions in GMP alone precipitate in several hours and re-form upon shaking; however, dispersions in the binary GMP/Guo gels are indefinitely stable. Increasing GMP or KCl concentration in the binary gels increased the relative abundance of large diameter and semi-conducting SWNTs. Different gel compositions also displayed different selectivities toward SWNTs of different chiralities. These results indicate a strong connection between the self-assembled G-gels and the dimensions and structures of the SWNTs that they solubilize.

## 1. Introduction

**Figure 1:**
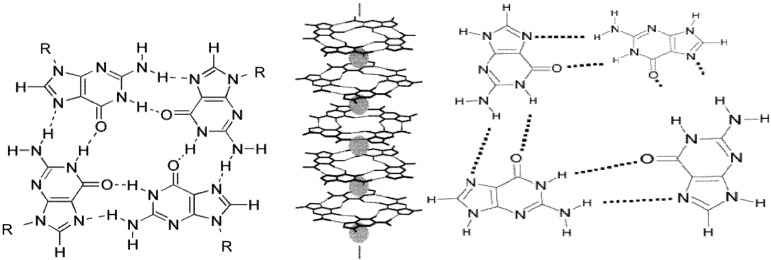
Self-assembly of guanosine compound monomers to form a planar guanosine tetrad (**left**), a columnar aggregate of guanosine tetrads stabilized by cations (gray spheres) centrally located between tetrads (**middle**), and a helical aggregate of hydrogen-bonded GMP monomers (**right**).

The unique aggregation behavior of guanine nucleosides and nucleotides has attracted interest in chemistry, biology, medicine and nanotechnology [1,2,3,4,5,6,7,8,9,10,11,12]. These compounds have a natural tendency to reversibly self assemble in aqueous solution into higher-order, organized structures through the formation of G-tetrads, in which each of four guanines is connected to its two nearest neighbors through Hoogsteen (G:G) hydrogen bonds (Figure 1, left). As monomer concentration increases, the G-tetrads form columnar, helical aggregates; these may be stabilized by metal cations (particularly K+) located centrally between adjacent tetrads and coordinated to the four oxygen atoms in each of the two tetrads (Figure 1, center). Alternatively, as in the case of 5'-guanosine monophosphate (GMP), the nucleotides may be arranged in a continuous helix through Hoogsteen hydrogen bonding interactions (Figure 1, right) [11,12]. In either case, as monomer concentration continues to increase, the columnar aggregates further assemble to form highly organized phases such as cholesteric and hexagonal liquid crystalline “G-gel” phases.

Until recently, G-quadruplex gelation had been investigated only in solutions containing a single guanosine compound. This changed with our discovery that binary mixtures of the soluble GMP and insoluble guanosine (Guo) in aqueous solution readily form gels over wide ranges of pH and temperature [13]. For example, at neutral pH and room temperature, GMP alone is too soluble in water to form gels, while Guo is too insoluble to enter the solution phase. By combining the two compounds, we were able to achieve highly tunable gelation that is a function of the proportion of the two guanosine compounds as well as pH, ionic strength and cation composition, although stable gels can form even in the absence of additional cations. A second study of the same system has been reported [14], as well as another binary system that combines Guo with a non-gelating guanosine derivative to form stable gels in aqueous solution [15].

**Figure 2:**
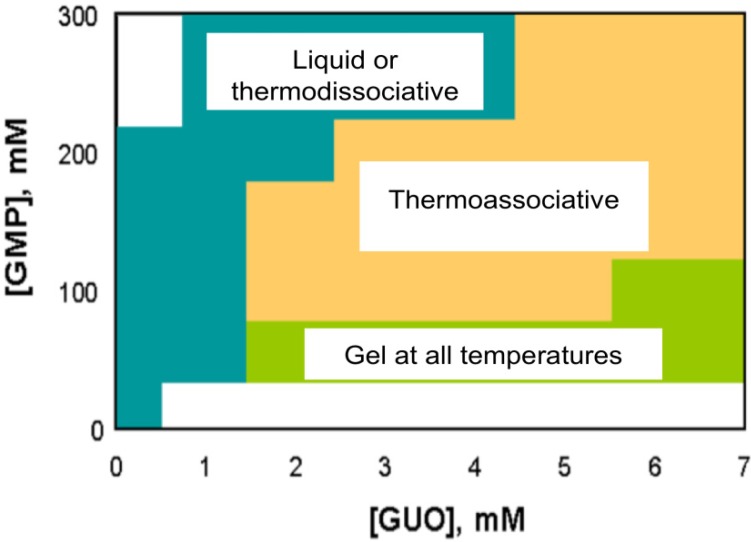
Phase diagram of binary G-gels as a function of GMP and Guo concentrations in 25 mM Tris buffer, pH 7.2, with 50 mM KCl.

A particularly intriguing property of the “binary” G-gels formed by GMP and Guo is the dramatic effect of the proportion of the two guanosine compounds on the thermoresponsiveness of the gels [13]. In some proportions, the binary gels are “thermodissociative”, *i.e.*, they gel with decreasing temperature, as is typical of solutions of individual guanosine compounds. However, in other proportions the binary gels exhibit the opposite, “thermoassociative” behavior, *i.e.*, they are liquids at low temperatures and form gels at higher temperatures. The thermal responsiveness of the solutions as a function of GMP and Guo concentrations is shown in Figure 2. In all cases, there is some temperature above which the G-quadruplex structure is completely disrupted and the organized liquid or gel phases “melt”.

Here we describe the discovery that the binary GMP/Guo gels are able to solubilize high concentrations of single-walled carbon nanotubes (SWNTs). Dispersion of SWNTs in most solvents, including water, is notoriously difficult. Because of commercial interest in SWNTs, intensive effort has been devoted to development of methods for dispersion of individual SWNTs in aqueous solution [16,17,18,19,20,21,22,23,24,25,26,27,28,29]. Common chemical methods such as covalent modification, strong acid treatment or fluorination that functionalize the SWNT surface have drawbacks such as breakage, introduction of defects, and irreversible alteration of SWNT structure that may result in loss of desirable properties. Physical methods that use various molecules or macromolecules such as DNA, peptides, synthetic polymers, detergents or organic molecules to coat or wrap the tubes offer nondestructive, potentially reversible solubilization of SWNTs; however, the strong tendency of SWNTs to be solubilized in bundles rather than as individual tubes often necessitates lengthy ultracentrifugation that may introduce breakage and defects. Solubilization of SWNTs in lipophilic guanosine derivatives in chloroform solution was recently reported [30]. In that work, dispersion of individual tubes was attributed to self-assembly of the guanosine derivatives into ribbon-like supramolecular structures.

In the present work, SWNTs are readily taken up by the aqueous G-gels as individual tubes, not in bundles, forming highly stable solutions with little or no sonication or centrifugation. We investigated SWNT solubilization in both thermodissociative and thermoassociative binary G-gels, in GMP alone, and for comparison, in 1% SDS. This work is relevant not only because of the potential applications for SWNTs in nanotechnology, but also because of the potential insight into the structure and assembly of G-quadruplex gels that may be achieved by studies of their interactions with SWNTs.

## 2. Results and Discussion

### 2.1. SWNT Solubilization

**Figure 3:**
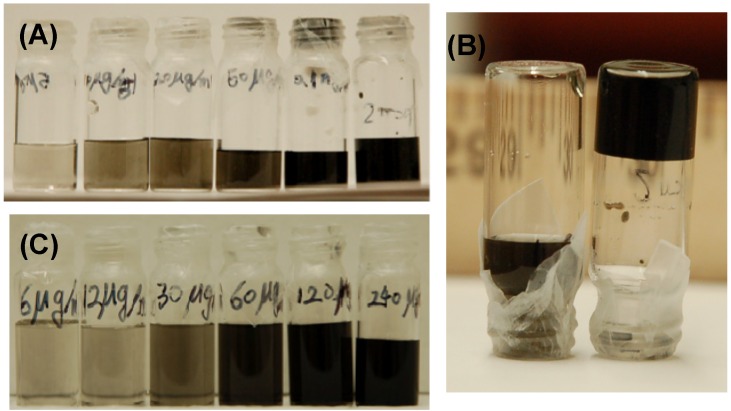
HiPco SWNT suspensions: (**A**) From left to right, 5, 10, 20, 50, 100 and 2,000 µg/mL SWNT in a thermodissociative gel containing 0.020 M Guo and 0.25 M GMP; (**B**) inverted vials containing 50 µg/mL SWNT (left, solution at bottom) and 2 mg/mL SWNT (right, gel suspension at top) in same gel as in A; (**C**) From left to right, 6, 12, 30, 60, 120 and 240 µg/mL SWNT in a thermoassociative gel containing 0.06 M Guo and 0.30 M GMP. Both G-gels were prepared in 25 mM Trizma buffer, pH 7.2 with 50 mM KCl.

Figure 3A shows HiPco SWNTs in a thermodissociative G-gel formed by 0.020 M Guo and 0.25 M GMP in 25 mM Trizma buffer, pH 7.2 with 50 mM KCl. Solutions containing as much as 5 mg/mL SWNT in this gel showed no visible signs of precipitation after 4 weeks. As shown in Figure 3B, solutions of low SWNT concentrations (<1 mg/mL) were liquid at room temperature while solutions of higher SWNT concentrations were firm gels. It was found that the latter gels exhibited thermoassociative behavior, becoming liquids upon refrigeration. Figure 3C shows SWNTs in a thermoassociative G-gel formed by 0.06 M Guo and 0.30 M GMP in the same buffer as the previous gel. This gel could solubilize several mg/mL SWNTs, again with no visible sign of precipitation after 4 weeks. The gelation temperature of the gel decreased from 33 °C in the absence of SWNT to lower temperatures with increasing SWNT until, above 2 mg/mL SWNT, the solution remained a firm gel even at 2 °C. Recovery of SWNTs from both gels could be achieved by heating the solution above the temperature at which the G-quadruplex structures completely melt, causing the SWNTs to precipitate. In contrast to the binary gels, a solution of 0.25 M GMP without Guo could readily solubilize 1–2 mg/mL SWNT but the SWNTs precipitated from solution over the course of 24 h. The dispersion could be re-formed by shaking the solution. SWNTs could not be suspended in Guo alone since Guo is poorly soluble in water and would not enter the aqueous solution with or without SWNTs.

We visually explored the effects of HiPco SWNTs on the gelation of a thermoassociative, binary G-gels formed by 0.25 M GMP and 0.06 M Guo in water with varying concentrations of KCl at low (refrigerator) temperature and room temperature. Solutions were visually classified as “liquid”, “viscous”, or “gel”. The term “liquid” describes a solution with water-like viscosity, the term “viscous” describes solutions that have taken on some gel-like thickness but can still move when the container is inverted, and the term “gel” describes firm gels that maintain their shape with no evidence of flowing when the container is inverted. The results are summarized in Table 1. In the absence of SWNT this thermoassociative gel is liquid at low temperature at all KCl concentrations and the gelation temperature increases from below room temperature at low KCl to above room temperature at high KCl. Addition of 1.2–2.8 mg/mL SWNT causes the gelation temperature of all of the solutions to decrease. This may be due to the high aspect ratio of SWNTs, which may provide a scaffold for gel formation in the binary G-gels and thereby facilitate organization and decrease the gelation temperature. In this regard, it should be noted that the dimensions of SWNTs are similar to those of the columnar aggregates formed by the G-tetrads that were depicted in Figure 1.

**Table 1:** Visual observation at Low Temperature (LT) and Room Temperature (RT) of the gelation of binary G-gels (0.25 M GMP, 0.06 M Guo, prepared in water with varying KCl), with and without HiPco SWNTs.

	Without SWNTs		With SWNTs		
KCl (M)	LT	RT	SWNT (mg/mL)	LT	RT
0.01	Liquid	Viscous	1.5	Gel	Gel
0.03	Liquid	Gel	1.2	Gel	Gel
0.07	Liquid	Viscous	2.1	Viscous	Gel
0.09	Liquid	Viscous	2.4	Liquid	Gel
0.15	Liquid	Liquid	2.8	Liquid	Viscous

### 2.2. Imaging Results

**Figure 4:**
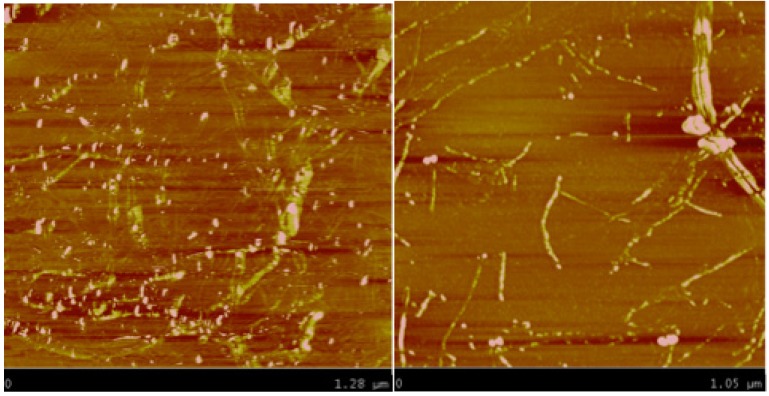
AFM images of 2 mg/mL SWNT in a thermodissociative gel containing 0.020 M Guo and 0.25 M GMP (**left**), and 240 µg/mL SWNT in thermoassociative gel containing 0.06 M Guo and 0.30 M GMP (**right**), both prepared in water with no KCl. X-axes are 1.3 µm (**left**) and 1.0 µm (**right**) full scale.

**Figure 5:**
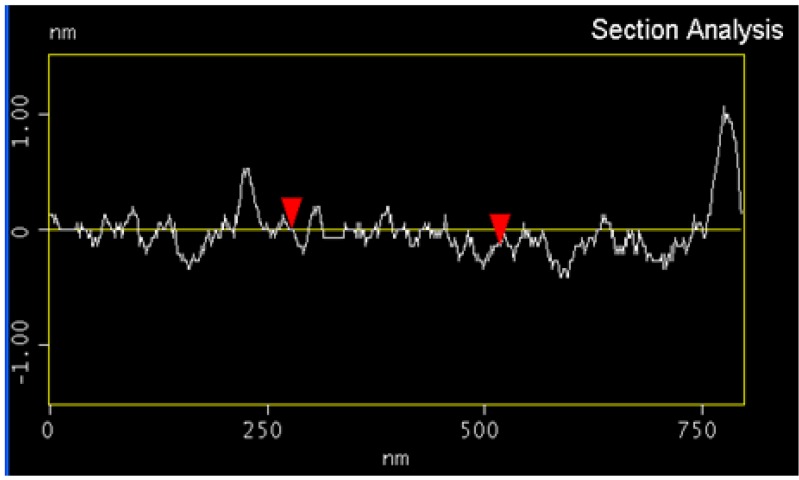
Line scan analysis of SWNTs in AFM image in Figure 4 left.

**Figure 6:**
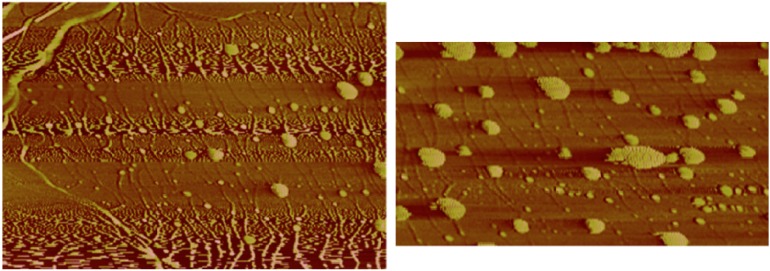
AFM image of HiPco SWNTs (0.6 mg total SWNT added per mL solution) in a thermoassociative binary G-gel containing 0.1 M GMP and 0.02 M Guo in water without KCl. The left image has a 4 µm full-scale x-axis. The right image is an expanded scale portion of the same image with a 1 µm full-scale x-axis.

Atomic force microscopic (AFM) images of the solutions of HiPco SWNTs from Figure 3 are shown in Figure 4. The image of 2 mg/mL SWNT in the thermodissociative gel (Figure 4, left) shows SWNT-like structures amid thicker features that may be due to large SWNT bundles, gel-coated SWNTs, or to the gel itself. The image of 240 µg/mL SWNTs in the thermoassociative gel without centrifugation (Figure 4, right) is dominated by well-dispersed SWNTs, with the exception of what appears to be a thick SWNT bundle in the upper right corner. Line scan analysis was performed on the regions containing individual SWNTs (Figure 5) and showed heights of in the range of 0.5–2 nm, which is consistent with SWNT diameters. A different sort of image with a higher degree of organization was observed for SWNTs in a thermoassociative gel formed by 0.10 M GMP and 0.02 M Guo in water with no KCl (Figure 6). Discrete structures such as those observed in Figure 4 and Figure 6 were not observed in images of the same gel solutions without SWNTs (not shown), although it is of course impossible to rule out the presence of such structures beyond the areas of the AFM images. The AFM results do suggest that SWNT dispersion varies as a function of gel composition.

### 2.3. Spectroscopic Measurements

**Figure 7:**
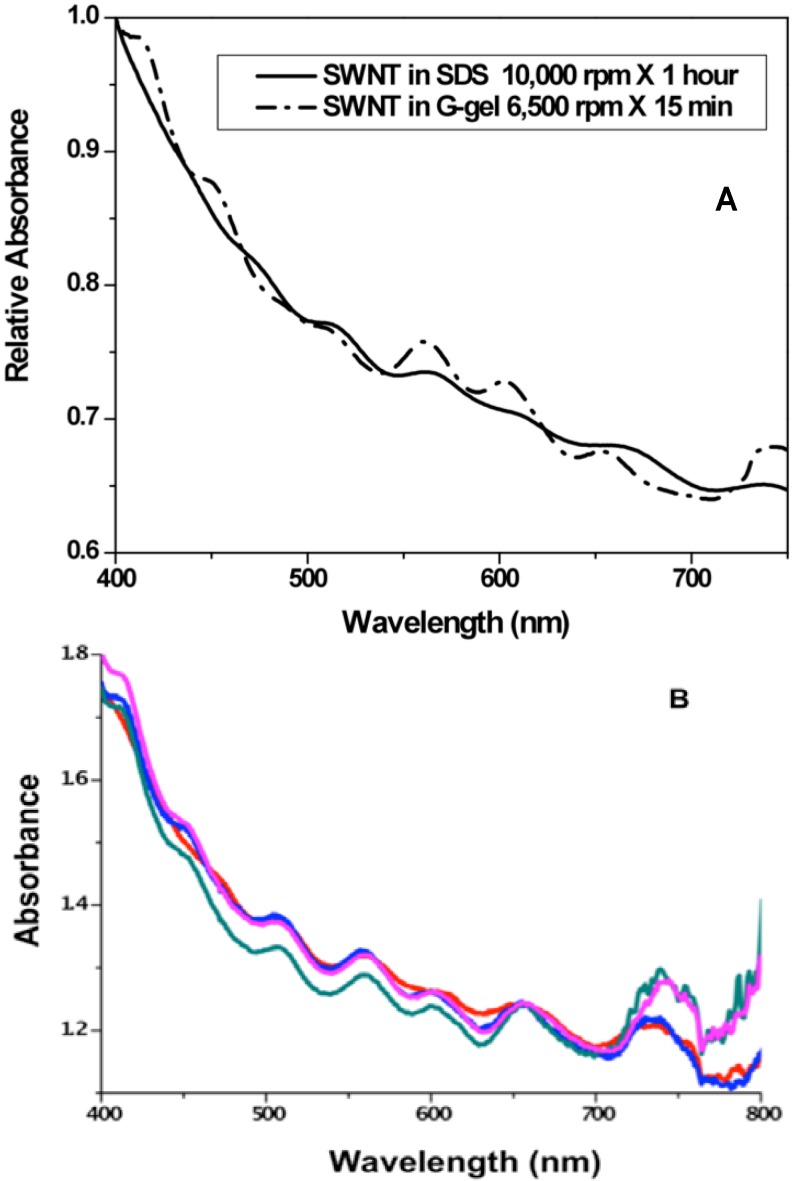
Absorption spectra of HiPco SWNTs in different media. (**A**) Spectra of SWNTs, normalized to the maximum absorbance at 400 nm, in 1% SDS after centrifugation at 10,000 rpm for 1 h and in a thermoassociative G-gel (0.25 M Guo and 0.03 M GMP in water without KCl) after centrifugation at 6,500 rpm for 15 min; (**B**) Spectra of SWNTs in the SDS solution after ultracentrifugation at 30,000 rpm for 3 hs and in G-gels containing 0.25 GMP (blue), 0.25 M GMP/0.01 M Guo (green) and 0.25 M GMP/0.04 M Guo (pink), after centrifugation at 9,000 rpm for 1 h.

The solubilization of individual SWNTs rather than bundles in the G-gels is demonstrated by UV-visible absorbance spectra. The absorbance spectra of SWNTs are known to be more highly resolved when the tubes are individually dispersed than when they are bundled since bundling can interfere with the electronic states of neighboring SWNTs, resulting in spectral broadening and red-shifting [29]. As shown in Figure 7A, the absorption spectrum of SWNTs in a binary G-gel measured after brief, low speed centrifugation exhibits better peak resolution than the spectrum of the SWNTs in 1% SDS measured after longer, higher speed centrifugation. Figure 7B compares SWNTs in SDS after lengthy ultracentrifugation with SWNTs in three different G-gels after briefer, low speed centrifugation. The SWNT dispersion in SDS is similar to those in the G-gels, except for higher absorbance above 700 nm in the binary G-gels compared to either SDS or GMP. This indicates that the binary G-gels solubilize a relatively higher abundance of larger diameter tubes.

**Figure 8:**
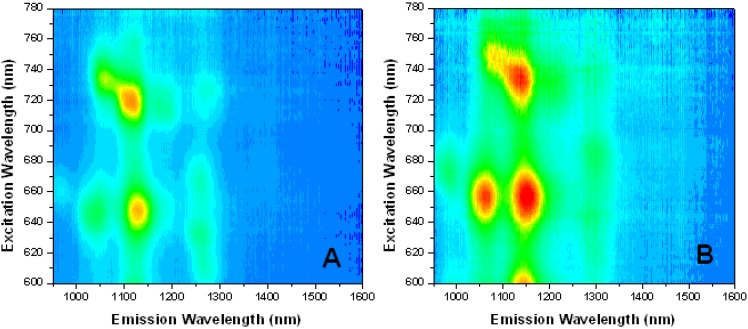
NIR fluorescence spectra of HiPco SWNTs in (**A**) 1% SDS after 1 h × 10,000 rpm centrifugation, and (**B**) 0.25 M GMP in water without KCl without centrifugation.

Figure 8 compares the Near-IR (NIR) fluorescence excitation-emission spectra of high-purity HiPco SWNTs in 1% SDS after centrifugation with SWNTs in 0.25 M GMP without centrifugation. The spectra inform us only about semiconducting SWNTs, since metallic SWNTs do not contribute to the NIR fluorescence. Each peak corresponds to a different chirality of semiconducting tube. Since emission intensity is much greater for individually dispersed SWNTs than for bundled tubes, the fluorescence results can be used to compare the solubilization of the semiconducting tubes in different media. The higher intensity of the SWNT emission in the G-gel compared to SDS in Figure 8 indicates a greater degree of dispersion of individual SWNTs. This is supported by the red shifts in the emission maxima, which suggest that the SWNTs are more closely associated with the gel structures compared to SDS [30] and experience a more hydrophobic environment [31], possibly due to pi-pi interactions between the guanosine moiteies in the gel and the aromatic groups on the SWNT surface.

**Figure 9:**
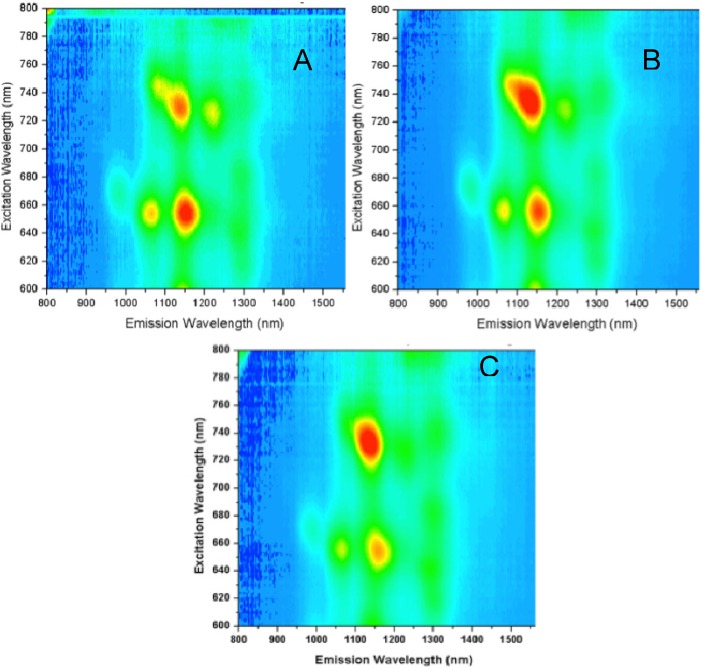
NIR fluorescence spectra of HiPco SWNTs in: (**A**) 0.25 M GMP, (**B**) 0.25 M GMP/0.01 M Guo, (**C**) 0.25 M GMP/0.04 M Guo, all prepared in water without KCl and briefly ultracentrifuged. Excitation wavelength (y-axis) is 600–800 nm and emission wavelength (x-axis) is 800–1,600 nm. The z-axis shows increasing intensity from blue to red, on scales of (**A**) 1.5–550, (**B**) −1.7 to 1200, and (**C**) −1.6 to 800.

Figure 9 shows NIR fluorescence spectra of HiPco SWNTs dispersed in G-gels formed by 0.25 GMP alone and in mixtures with 0.01 M and 0.04 M Guo. These solutions were briefly ultracentrifuged. There are pronounced differences in relative peak intensities among the three different solutions, indicating that the three media have different selectivities toward the various chiral SWNTs.

**Figure 10:**
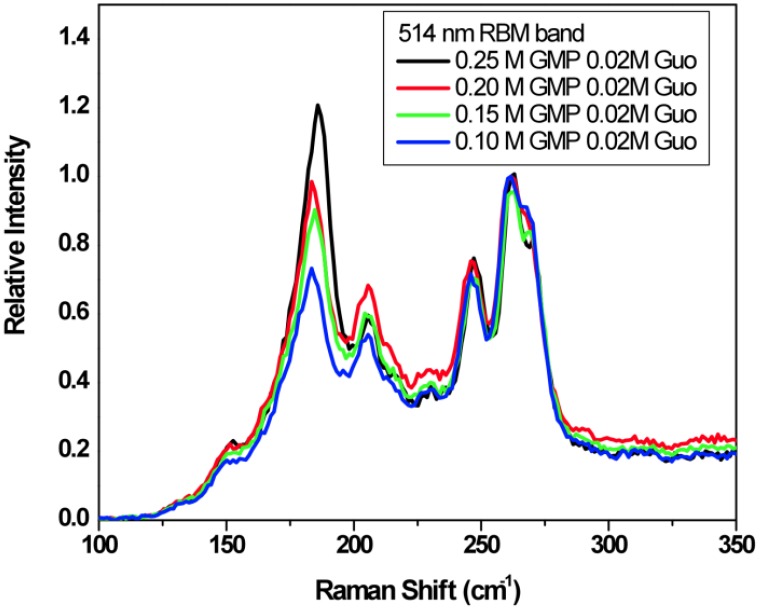
The RBM region Raman Spectra of SWNTs in binary G-gels containing 0.02 M Guo and various GMP concentrations in water without KCl. The spectra were excited at 514 nm and normalized to the 262 cm^−1^ peak.

**Figure 11:**
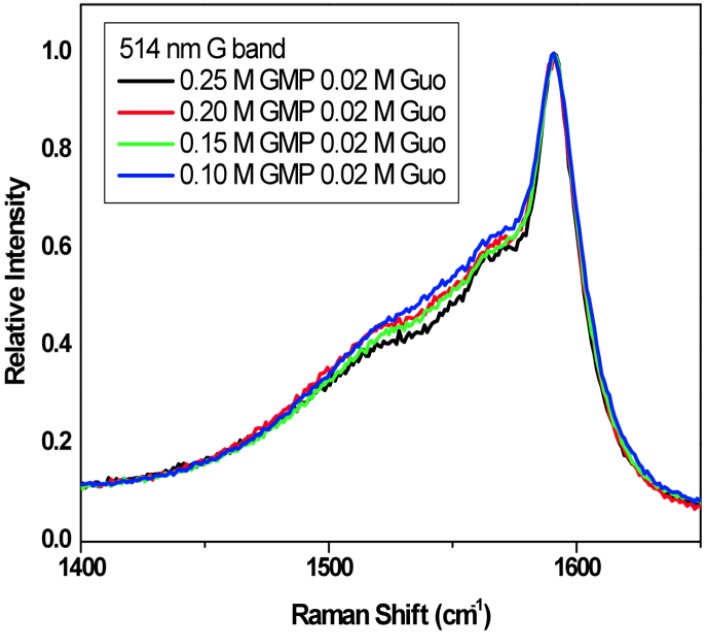
The G-band Raman spectra of the same solutions as in Figure 10. The spectra were excited at 514 nm and normalized to the 1580 cm^−1^ peak.

Figure 10 shows the Radial Breathing Mode (RBM) region of the Raman spectra for SWNTs in binary gels containing 0.02 M Guo with varying GMP concentration from 0.10 M to 0.25 M. The spectra were acquired using the 514 nm laser line, which excites both metallic and semiconductor SWNTs. According to the literature [32], the peaks at 186 and 206 cm^−1^ correspond to semiconducting SWNTs and the peaks at 230, 248, 262 and 271 cm^−1^ correspond to metallic SWNTs. Increasing GMP in the G-gel results in an increase in the relative signal from the semiconducting nanotubes, indicating increasing selectivity towards the semiconducting tubes, or increasing selectivity toward larger diameter tubes since decreasing Raman shifts in the RBM region correspond to increasing diameter tubes. Figure 11 shows the microRaman spectra in the tangential mode G-band region of the Raman spectra. The spectra were normalized to the peak at 1580 cm^−1^, which is mostly from semiconducting nanotubes. The decrease in the relative intensity of the broad region at shorter spectral shifts, which is mostly from metallic nanotubes, again indicates that increasing GMP relative to Guo in the gels increases the relative abundance of semiconducting nanotubes.

## 3. Experimental

### 3.1. Materials

All chemical reagents and buffers, Guanosine (Guo > 98%), 5'-Guanosine monophosphate disodium salt (GMP > 99%) and sodium dodecyl sulfate (SDS) were from Sigma Aldrich (St. Louis, MO, USA). Super purified high pressure CO conversion single walled carbon nanotubes (HiPco SWNTs) that were purified to remove large catalyst particles and <5 wt% ash content were from Carbon Nanotechnology (Houston, TX, USA). Water was purified using ultrapure Milli-Q from Millipore (Bedford, MA, USA).

### 3.2. Gel Preparation

Binary G-gels were prepared in 25 mM Tris buffer (pH 7.2) or in ultrapure water, with 0–0.15 M KCl. The appropriate amounts of Guo and 5'-GMP were dissolved in buffer under stirring and heated for 3 to 5 min at 80 °C. After all chemicals were dissolved, the solution was cooled to room temperature with stirring and then refrigerated overnight at 4 °C prior to use. In the spectroscopic studies, the solutions were stored in the refrigerator at all times except when in use.

### 3.3. Atomic Force Microscopy

AFM images were collected at room temperature in the tapping mode using a Multimode Scanning Probe Microscope from Digital Instruments (Plainview, NY, USA) with a Mikromash DP15/GP tip (San Jose, CA, USA). The scan speed was set at 0.5 Hz with a scan step of 1–4 µm. AFM samples were prepared by simply dropping SWNT solutions onto a Si substrate without spin coating. The droplet was applied under gentle shaking to ensure a thin layer and dried at different temperatures depending on the particular experiment. The collected images were processed using software provided by the instrument manufacturer to get line scan information and improve image contrast.

### 3.4. UV-Visible Absorption Spectroscopy

Absorbance spectra were collected using an Agilent 8453 UV/Vis absorption spectrometer (Santa Clara, CA, USA) equipped with a 150 W lamp source. All SWNT solutions were contained in 5 mm pathlength quartz cuvettes and analyzed at 25 °C using a 1 nm monochromator slit width. Blank solutions consisting of SDS or G-gel without SWNTs were measured in the same cuvette prior to measurement of the SWNT sample and the blank spectrum subtracted from the sample spectrum.

### 3.5. Near-Infrared Fluorescence Spectroscopy

Near-infrared (NIR) fluorescence spectra of SWNT solutions were measured using a Fluorolog-3 spectrometer on-site at Horiba Jobin-Yvon (Edison, NJ, USA). The spectrometer was equipped with a liquid nitrogen cooled CCD array as detector and a 450 W xenon arc lamp. Due to the high concentration of the SWNTs in the binary G-gels, fluorescence was measured in front face mode, in which the emission photons are collected at the front face at an angle of approximately 22.5° relative to the excitation beam. The samples were contained in 1 cm pathlength quartz fluorescence cuvettes. The excitation wavelength ranged from 600 nm to 800 nm and the emission wavelength range was 800 nm to 1,600 nm. All samples are measured at room temperature.

### 3.6. MicroRaman Spectroscopy

Raman spectra were measured using a ReniShaw MicroRaman Scope 2000 (Wotton-under-Edge, Gloucestershire, UK). In all of the microRaman experiments, the SWNT solutions were rinsed three times with water and ethanol to remove the gel, resuspended in ethanol, and dried on a Si substrate. The spectra were obtained using the 514 nm (2.1 eV) laser line, which excites both semiconducting and metallic SWNTS. Each spectrum is the average of 6 to 10 individual spectra collected from randomly chosen spots in the sample and normalized to one of the peaks as indicated in the figure captions.

## 4. Conclusions

Experience with agents such as organic polymers, biopolymers and surfactants has led to general acceptance that SWNTs are solubilized primarily in bundles and that lengthy ultracentrifugation is needed to achieve solutions of individually dispersed SWNTs. In the present work, the ease with which G-gels formed by mixtures of GMP and Guo solubilize SWNTs at high concentrations with simple mixing to form highly stable solutions of individually dispersed and, in some cases, aligned SWNTs indicates that binary G-gels act through a different mechanism of solubilization, one in which the SWNTs are directly solubilized as individual tubes rather than as bundles. The spectral shifts are consistent with an increase in the hydrophobicity of the SWNT surface environment, which suggests that the solubilization mechanism involves pi-pi interactions between the guanines in the G-gel and the aromatic rings on the SWNT surface [30,31].

The presence of both GMP and Guo is essential to the formation of stable SWNT solutions, indicating that the binary G-gels interact with SWNTs through a mechanism that is not available to G-gels formed by GMP alone. It was also observed that SWNTs promoted gelation and decreased the gelation temperature of thermoassociative G-gels, and that increasing the GMP:Guo ratio or KCl increased the relative abundance of larger diameter and/or semiconducting tubes in the solutions. Different G-gel compositions were also found to have different relative abundances of semiconductor tubes of different chiralities. The selectivity of different G-gels toward different SWNT structures is further evidence of individual solubilization of the SWNTs, since such selectivity would not be expected if the tubes were primarily in large bundles. These results show promise for G-quadruplex gels in solubilization of SWNTs and fractionation of tubes based on diameter, conductive properties and chirality. They also suggest that SWNTs could be used to gain insight into the structure and self-assembly of G-gels. This is significant for development of G-gels as biomaterials and for studies of prebiotic chemistry, in which the formation of organized G-gel phases that selectively solubilize or include nanostructures of like dimensions could play a role in formation of early polymers.
